# 2465. O-serotype Distribution of *Escherichia coli* Causing Invasive Disease in Tertiary Care Hospital Patients

**DOI:** 10.1093/ofid/ofad500.2083

**Published:** 2023-11-27

**Authors:** Jeroen Geurtsen, Joachim Doua, Luis Martinez-Martinez, Patricia Ibarra de Palacios, Jeff Powis, Matthew Sims, Peter Hermans, Oliver Barraud, Philippe Lanotte, Joshua T Thaden, Oscar Go, Bart Spiessens, Darren Abbanat, Florian Wagenlehner, Tetsuya Matsumoto, Marc Bonten, Michal Sarnecki, Jan Poolman

**Affiliations:** Janssen Vaccines & Prevention BV, Leiden, Zuid-Holland, Netherlands; Janssen Research & Development, Infectious Diseases & Vaccines, Janssen Pharmaceutica, Beerse, Antwerpen, Belgium; Hospital University Reina Sofia; University of Cordoba, Cordoba, Andalucia, Spain; Janssen Vaccines, Bern, Bern, Switzerland; University of Toronto, Toronto, ON, Canada; William Beaumont University Hospital, Corewell Health, Royal Oak, Michigan; Julius Center for Health Sciences and Primary Care, University Medical Center, Utrecht, Utrecht, Netherlands; CIC1435, CHU Limoges, Limoges, Limousin, France; Bretonneau Hospital, Tours University Hospital Centre, University of Tours-INRAE, Tours, Centre, France; Duke University School of Medicine, Durham, North Carolina; Janssen Research & Development, Raritan, New Jersey; Janssen Research & Development, Infectious Diseases & Vaccines, Janssen Pharmaceutica, Beerse, Antwerpen, Belgium; Janssen Research & Development, Raritan, New Jersey; Justuf Liebeg University Diessen, Diessen, Hessen, Germany; International University of Health and Welfare, Narita-shi, Chiba, Japan; Julius Center for Health Sciences and Primary Care, University Medical Center, Utrecht, Utrecht, Netherlands; Janssen Research & Development, Janssen Vaccines, Bern, Bern, Switzerland; Bacterial Vaccines Discovery and Early Development, Janssen Vaccines & Prevention B.V., Leiden, Zuid-Holland, Netherlands

## Abstract

**Background:**

*Escherichia coli* is a common Gram-negative bacterium that can infect normally sterile body sites and cause invasive *E. coli* disease (IED) including bacteremia, sepsis and septic shock. *E. coli* surface O-antigens are important virulence factors that contribute to pathogenicity, making them promising targets for the development of multivalent conjugate vaccines to protect against IED. Here, we describe the prevalence of O-serotypes and O-genotypes of clinical *E. coli* isolates across a multinational cohort of patients with IED.

**Methods:**

This was a retrospective, multicenter, noninterventional study across 17 tertiary care hospitals in Europe, North America and Asia. Patients with an IED diagnosis in the 12 months prior to data collection were included. IED was defined as *E. coli* presence in cultures from any normally sterile body site or urine in patients exhibiting clinical criteria of invasive disease (i.e., systemic inflammatory response syndrome [SIRS], sepsis, or septic shock) and no other identifiable site of infection. O-serotyping (agglutination) and O-genotyping (whole genome sequencing [WGS]) were conducted. Subgroup analyses were performed in isolates from patients with bacteremic vs nonbacteremic IED and in patients ≥60 years old.

**Results:**

902 patients with IED were identified (median age at initial IED diagnosis, 71.0 years; 51.6% male). The most common O-serotypes (prevalence ≥5%) based on O-genotyping were O25 (17.3% [95% CI, 14.82–20.06%]), O2 (11.7% [95% CI, 9.61–14.08%]), O6 (9.3% [95% CI, 7.44–11.49%]), O1 (6.3% [95% CI, 4.78–8.20%]), O15 (5.3% [95% CI, 3.85– 6.99%]) and O75 (5.0% [95% CI, 3.64–6.72%]) (**Table 1**). Collectively, these 6 most prevalent serotypes accounted for 55.0% of total isolates. A similar pattern of O-serotypes was observed in the subgroup of patients ≥60 years old (**Table 2**), with serotypes O25, O2 and O6 most common in both bacteremic and nonbacteremic IED isolates.

**Figure:**
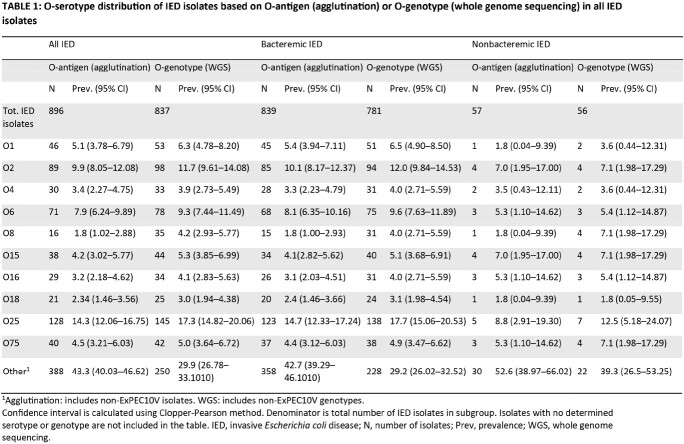

**Figure:**
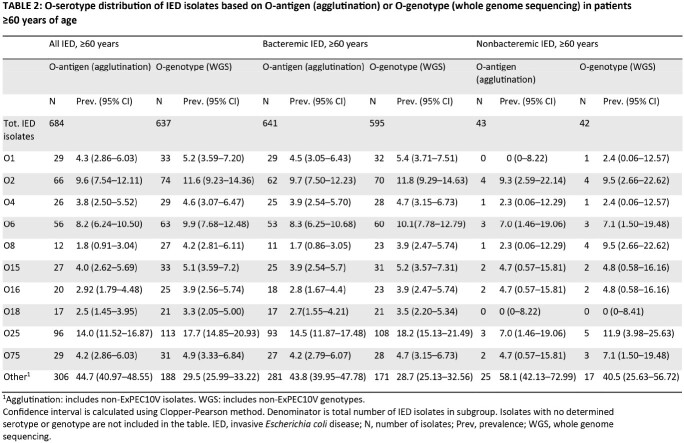

**Conclusion:**

The most predominant O-serotype among IED isolates from hospitalized patients with IED was O25, followed by O2, O6, O1, O15 and O75. Such epidemiological data could inform the development of an effective prophylactic vaccine against IED.

**Disclosures:**

**Jeroen Geurtsen, PhD**, Janssen: Employee|Janssen: Stocks/Bonds **Joachim Doua, MD, MPH**, Janssen: Employee|Janssen: Stocks/Bonds **Patricia Ibarra de Palacios, MD**, Janssen: Employee at the time of analysis **Matthew Sims, MD, PhD**, Astra-Zeneca: Investigator for company-sponsored studies|ContraFect: Investigator for company-sponsored studies|Crestone: Investigator for company-sponsored studies|Finch: Investigator for company-sponsored studies|Janssen: Investigator for company-sponsored studies|Leonard-Meron: Investigator for company-sponsored studies|Merck and Co: Investigator for company-sponsored studies|OpGen Inc: Advisor/Consultant|OpGen Inc: Investigator for company-sponsored studies|Pfizer: Investigator for company-sponsored studies|Prenosis: Advisor/Consultant|Prenosis: Investigator for company-sponsored studies|QIAGEN Sciences LLC: Investigator for company-sponsored studies|Roche: Investigator for company-sponsored studies|Seres Therapeutics: Investigator for company-sponsored studies **Peter Hermans, PhD**, Janssen: Employee at the time of analysis **Joshua T. Thaden, MD, PhD**, Resonantia Diagnostics, Inc: Advisor/Consultant **Oscar Go, PhD**, Janssen: Employee|Janssen: Stocks/Bonds **Bart Spiessens, PhD**, Janssen: Employee|Janssen: Stocks/Bonds **Darren Abbanat, PhD**, Janssen: Employee at the time of analysis **Florian Wagenlehner, MD**, Achaogen: Advisory Board member, study participation|Astellas: Honoraria|AstraZeneca: Honoraria|AstraZeneca: Advisory Board member|Biomedical Advanced Research and Development Authority (BARDA): Grant/Research Support|Bionorica: Honoraria|Bionorica: Meeting/travel support, study participation|Deutsches Zentrum für Infektionsforschung (DZIF): Study participation|Enteris BioPharma: Study participation|Everest Medicines: Grant/Research Support|German S3 guideline Urinary tract infections: Board Member|Glaxo Smith Kline: Advisor/Consultant|Glaxo Smith Kline: Honoraria|Glaxo Smith Kline: Consulting fees, meeting/travel support, advisory board member, principal investigator in a GSK-sponsored study|Global Antibiotic Research and Development Partnership (GARDP Foundation): Grant/Research Support|Guidelines European Association of Urology: Infections in Urology: Board Member|Helperby Therapeutics: Study participation|Janssen: Honoraria|Janssen: Advisory Board member|Klosterfrau: Honoraria|LeoPharma: Advisory Board member|MerLion: Advisory Board member|MIP Pharma: Honoraria|MSD: Advisory Board member|OM Pharma/Vifor Pharma: Advisory Board member, study participation|OM-Pharma: Honoraria|Pfizer: Honoraria|Pfizer: Advisory Board member|RosenPharma: Advisory Board member|Shionogi: Advisory Board member, study participation|Speaker research group German research foundation (DFG) Bacterial Renal Infections and Defense (FOR 5427): Study participation|Spero Therapeutics: Advisor/Consultant|Spero Therapeutics: Consulting fees|University Hospital Giessen and Marburg GmbH, and Justus Liebig University, Germany: Employee|Venatorx Pharmaceuticals, Inc.: Advisor/Consultant|Venatorx Pharmaceuticals, Inc.: Grant/Research Support|Venatorx Pharmaceuticals, Inc.: Consulting fees, Advisory Board member **Tetsuya Matsumoto, MD; PhD**, member of the international study steering committee for the E.mbrace study and reports payment: Board Member **Marc Bonten, MD, PhD**, chair of the international study steering committee for the E.mbrace study (Janssen Vaccines), with payments made to UMC Utrecht: Board Member **Michal Sarnecki, MD**, Janssen: Employee|Janssen: Stocks/Bonds **Jan Poolman, PhD**, Janssen: Employee|Janssen: Stocks/Bonds

